# Beamline P02.1 at PETRA III for high-resolution and high-energy powder diffraction

**DOI:** 10.1107/S1600577515002222

**Published:** 2015-04-14

**Authors:** Ann-Christin Dippel, Hanns-Peter Liermann, Jan Torben Delitz, Peter Walter, Horst Schulte-Schrepping, Oliver H. Seeck, Hermann Franz

**Affiliations:** a Deutsches Elektronen-Synchrotron DESY, Notkestrasse 85, D-22607 Hamburg, Germany

**Keywords:** synchrotron powder diffraction, high-energy X-rays, high angular resolution, time-resolved experiments, X-ray total scattering

## Abstract

Powder X-ray diffraction techniques largely benefit from the superior beam quality provided by high-brilliance synchrotron light sources in terms of photon flux and angular resolution. The High Resolution Powder Diffraction Beamline P02.1 at the storage ring PETRA III (DESY, Hamburg, Germany) combines these strengths with the power of high-energy X-rays for materials research. The beamline is operated at a fixed photon energy of 60 keV (0.207 Å wavelength). A high-resolution monochromator generates the highly collimated X-ray beam of narrow energy bandwidth. Classic crystal structure determination in reciprocal space at standard and non-ambient conditions are an essential part of the scientific scope as well as total scattering analysis using the real space information of the pair distribution function. Both methods are complemented by *in situ* capabilities with time-resolution in the sub-second regime owing to the high beam intensity and the advanced detector technology for high-energy X-rays. P02.1’s efficiency in solving chemical and crystallographic problems is illustrated by presenting key experiments that were carried out within these fields during the early stage of beamline operation.

## Abstract 2

By providing the capabilities for high-resolution, high-energy and time-resolved powder X-ray diffraction, beamline P02.1 is a versatile tool to tackle various problems in materials science, crystallography and chemistry.

## 1. Introduction   

Powder X-ray diffraction (PXRD) is one of the elementary and most powerful tools for studying the structure of materials. It is a widely used technique in the laboratory as well as at large-scale synchrotron facilities (see, for example, Dinnebier & Billinge, 2008 ▶; David *et al.*, 2006 ▶). In particular, the superior angular resolution, the highly intense X-ray beam and the wavelength tunability distinguish synchrotron PXRD beamlines from even the most modern laboratory instruments. These characteristics enable, for example, the analysis of materials with very complex crystal structure and mixtures of several compounds at different concentrations, the direct observation of structural changes at high time resolution under non-ambient conditions, high-throughput screening studies, as well as anomalous scattering at distinct absorption edges (see, for example, Stephens *et al.*, 2006 ▶). When the energy of the photon beam is high, *i.e.* ≥30 keV (corresponding to short wavelengths ≤0.41 Å), PXRD experiments may conveniently be performed in simple transmission geometry. Due to the relatively low absorption, reflections over a wide range in reciprocal space are measured to accurate intensities even for high-*Z* materials. In addition, the large penetration power of high-energy X-rays into bulky sample environments and reactors allows for studying materials *in situ* and *operando* under real conditions. Finally, as data up to high values of momentum transfer *Q* are available, it is possible to investigate the short-range order of amorphous, nanocrystalline and disordered materials. By means of total scattering analysis, *i.e.* combining reciprocal and real space information from the Bragg reflections and the diffuse scattering, the structure can be fully determined on different length scales.

At the high-brilliance third-generation storage ring PETRA III at the German research facility Deutsches Elektronen-Synchrotron (DESY) in Hamburg (Franz *et al.*, 2006 ▶), the High Resolution Powder Diffraction Beamline P02.1 combines the capabilities of high-resolution and high-energy PXRD. The beamline is operated at a fixed energy of 60 keV (wavelength 0.207 Å) with a relative energy bandwidth Δ*E*/*E* of the order of 10^−4^. While this energy spread is typical for high-resolution PXRD beamlines, it is very low for high-energy diffraction instruments that usually sacrifice resolution for increased photon flux (see, for example, Shastri, 2004 ▶; Kvick, 2003 ▶; Tschentscher & Suortti, 1998 ▶). Compared with other high-resolution powder diffraction beamlines at third-generation synchrotron light sources (see, for example, Fitch, 2004 ▶; Wang *et al.*, 2008 ▶; Ikeda *et al.*, 2003 ▶; Thompson *et al.*, 2009 ▶; Patterson *et al.*, 2005 ▶; Cerenius & Svensson, 2006 ▶; Wallwork *et al.*, 2007 ▶; Fauth *et al.*, 2013 ▶), P02.1 differs significantly with respect to the provided photon energy. While 60 keV is well within the available energy range at beamline ID22 (former ID31) at the European Synchrotron Radiation Facility (ERSF; Fitch, 2004 ▶), all other existing dedicated PXRD stations do not provide X-rays in this energy range. Among the upcoming beamlines at new facilities, *e.g.* NSLS-II in the USA (Shi *et al.*, 2013 ▶) and MAX IV in Sweden (Cerenius & Svensson, 2006 ▶), only the former will exploit the benefits of high-energy PXRD at high angular resolution.

This paper depicts the layout and performance of beamline P02.1. In particular, the capabilities for *in situ* and time-resolved studies are emphasized and illustrated by recent cutting-edge experiments. After completing the commissioning phase of the high-resolution setup that is currently in progress, a dedicated description will be published separately by Vad *et al.* (2015 ▶) which will include specifics of the diffractometer and multi-analyzer crystal detector design as well as their experimental performance.

## 2. Scope   

The High Resolution Powder Diffraction Beamline P02.1 provides an X-ray beam whose key parameters are high energy (short wavelength), high photon flux, as well as low angular divergence and small energy bandwidth. One or more of these features are essential for each of the different types of experimental techniques that are performed at P02.1. These can be grouped into three categories: (i) high-resolution PXRD to solve and refine crystal structures from powders, to perform quantitative phase analysis, and to evaluate the microstructure by line profile analysis, (ii) high-energy PXRD to determine the local structure of complex materials, *e.g.* by analysing the atomic pair distribution function in addition to their average structure (X-ray total scattering technique), and (iii) fast PXRD to study *in situ* transformations under non-ambient conditions. Moreover, the availability of an area detector facilitates the efficient examination of structural anisotropies in two-dimensional diffraction patterns. Thus, beamline P02.1 provides the tools to investigate structure features and aspects of *in situ* experiments that include:

• low-symmetry crystal structures, *e.g.* triclinic and monoclinic systems;
• high-*Z* materials exhibiting short attenuation lengths;
• mixtures of several phases and/or compounds;
• trace phases in the presence of a strong phase;
• texture and preferred orientation;
• tensile/compressive stress effects;
• local disorder of the average crystal lattice;
• nano-sized structures and shapes, *e.g.* nanoparticles, nanorods;
• short-range order, *e.g.* in glasses and other non-crystalline solids;
• tracking of phase transitions under non-ambient conditions, *e.g.* thermal treatment;
• kinetics of structural changes, *e.g.* crystallization, decomposition;
• reaction intermediates and unstable phases with short lifetime;
• growth mechanisms of grains and layers;
• distortions induced by an external electric field, *e.g.* piezoelectric and ferroelectric materials.

Integrated sample environments for thermal treatment and automated sample changing systems are designed for high-throughput measurements under standardized conditions. Furthermore, the flexible beamline layout with a large movable sample table and movable beamline optics (see §4) facilitates the installation of sample holders and bulky or heavy *in situ* setups that are customized for user-specific experiments. The high X-ray energy of 60 keV provides effective penetration of enclosures of sealed sample cells or reactors made from polymers, aluminum or even steel. Depending on the complexity of the specimen structure and the time-scale of the transformations to be studied, data are collected either on the high-resolution detector (under development) or the fast and large-area detector (§5).

## 3. Optics   

### 3.1. Undulator source and photon energy   

**Figure 1:**
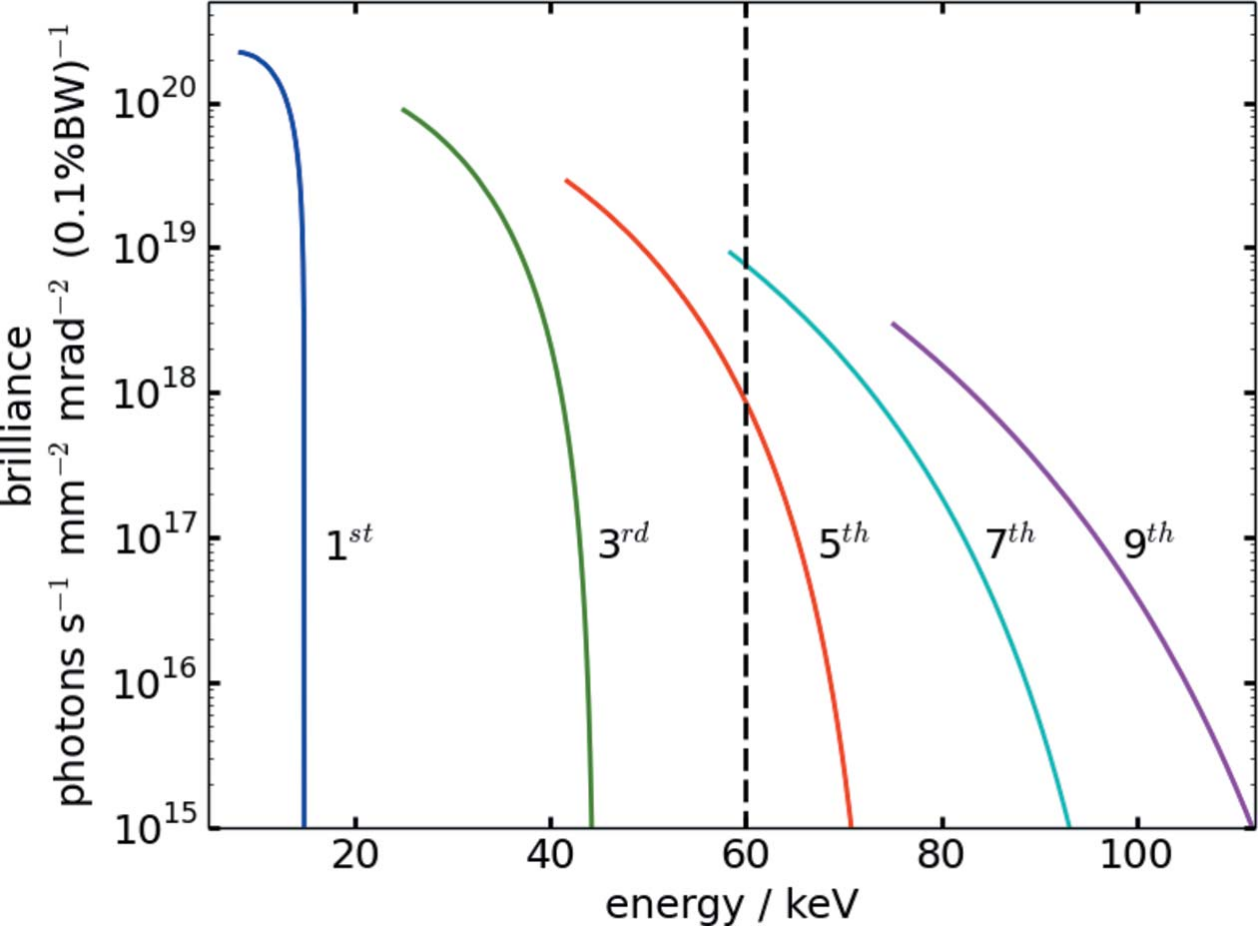
Undulator spectrum of U23 showing the first five odd harmonics. The black dashed line indicates the operation energy of 60 keV.

Beamline P02.1 is operated as the side branch of the Hard X-ray Diffraction Beamline P02 with the Extreme Conditions Beamline P02.2 (Liermann *et al.*, 2015 ▶) as the inline station. The common source for both beamlines is an undulator specifically designed to generate high-energy X-rays. While high photon energies are a prerequisite for experiments under extreme conditions at P02.2, they are particularly useful for powder diffraction techniques as well, as described in the previous sections. As a consequence, it was decided to operate P02.1 at a relatively high X-ray energy while maintaining Δ*E*/*E* in the order of 10^−4^ at maximum flux. Owing to the high particle energy and the high brilliance of PETRA III, X-ray energies >50 keV were viable. Additional consideration was given to the reduction of fluorescence originating from beamline components made from tungsten, such as slit systems, by selecting a beam energy below the W *K*-edge of 69.5 keV. The choice of the undulator design parameters was based on the desired high photon energy in the range of 60 keV and the geometrical boundary conditions on the sector to accommodate a side branch. The resulting undulator type called U23 (Barthelmess *et al.*, 2008 ▶) has a total length of 2 m and a period length of 23 mm. Fig. 1 ▶ depicts the brilliance spectrum characteristic for the radiation produced by undulator U23. In Tables S1 and S2 of the supporting information, machine and device parameters relevant for the calculation of the undulator spectrum are summarized. Maximum flux is available on the low-energy end of each harmonic by closing the undulator to the smallest magnetic gap. Simultaneous operation of both beamlines is realized by keeping the undulator gap constant. In the chosen configuration, beamline P02.1 is operated on the seventh harmonic at 60 keV (marked by the dashed line in Fig. 1 ▶), while the inline station selects either of the third to the ninth harmonic, yielding 25.6, 42.7, 60.0 or 77.1 keV, respectively.

### 3.2. Monochromator   

#### 3.2.1. Conceptual design   

**Figure 2:**
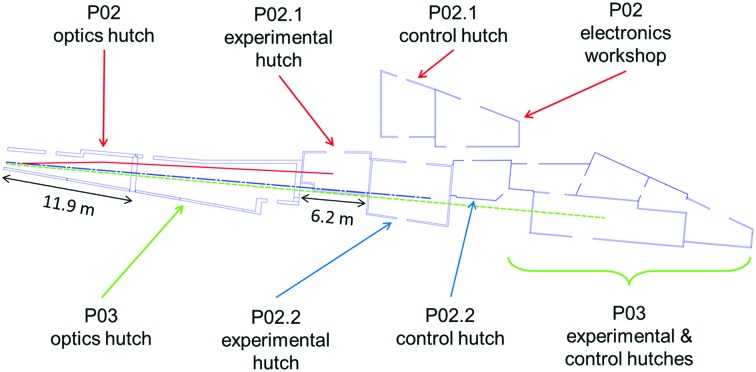
Floor plan of sector 2 and adjacent P02 hutches with beam paths. Beam path of P02.1 is shown in red (full line), P02.2 in blue (dot-dashed) and P03 in green (dashed).

In general, geometrical considerations of the sector layout play a crucial role when designing a side station. Beamlines P02 and P03 (Buffet *et al.*, 2012 ▶) share one sector in the experimental hall. The two corresponding undulators are installed in one straight section of the storage ring and are canted at an angle of 5 mrad. As can be seen from the floor plan given in Fig. 2 ▶, this arrangement results in a lateral offset of less than 15 cm between the P02 and P03 beam paths at the entry point to the P02 optics hutch. The presence of two back-to-back optics hutches in the sector ruled out the option of a single-bounce monochromator which can only provide beam to a station directly adjacent to the corresponding optics hutch. Consequently, a horizontally scattering double-crystal monochromator was installed in the first optics hutch. A special vacuum chamber was designed to integrate the first monochromator crystal for P02.1 in the available space. Mounted in a separate vacuum chamber, the second monochromator crystal redirects the beam roughly along the sector axis at a suitable lateral distance.

Both monochromator crystals diffract the X-ray beam horizontally in Laue (transmission) geometry, which offers three basic advantages over Bragg (reflection) geometry: (i) the absorption of the transmitted beam for P02.2 is lower by orders of magnitude, (ii) the required crystal size is significantly smaller since the very small Bragg angles involved at high photon energies produce a large footprint when reflected off the crystal surface, and (iii) the transmission geometry is less sensitive to small beam oscillations because the source point of diffraction along the beam (*i.e.* the thin crystal itself) stays the same as opposed to a wandering footprint in reflection geometry. The first crystal is preferably made of a low-*Z* material, *e.g.* diamond, in order to minimize absorption. Considering a 400 µm thin crystal, diamond attenuates the beam by less than 5% even at the lowest P02.2 operation energy of 25.6 keV as opposed to more than 18% in the case of an equivalent silicon crystal. In addition, diamond exhibits higher heat conductance than standard silicon monochromator crystals as well as higher Bragg angles, which facilitate achieving a suitable lateral offset between inline and side station at shorter distance along the beam. A detailed study evaluating different combinations of crystal materials, geometries and asymmetry angles was carried out by Vad *et al.* (2015 ▶). Taking into account all practical and theoretical considerations, the optimum combination for P02.1 is a diamond crystal as the first and a silicon crystal as the second monochromator, both employing 111 reflections. Owing to its high heat conductance, diamond can withstand the heat load deposited by the white beam from the undulator when applying water cooling instead of cryogenic cooling. The manufacturer *Element Six* provided the diamond crystal which has a thickness of 400 µm, roughness of 1 nm and an asymmetric cut angle of 54.74°, *i.e.* the angle between the (100) surface and the (111) diffraction planes. The holder for the diamond crystal consists of a bevelled copper block that orients the crystal platelet so as to pre-align the 111 reflection. Within a notch in this copper body, the crystal is held in place by a holed crossbar. The contact to the metal at the base and inside the hole is realized through the liquid alloy Galinstan^®^ that is highly heat conducting and minimizes mechanical stress. At a distance of approximately 8 m from the diamond crystal, the silicon crystal is set up in a similar stage without cooling. This crystal was cut symmetrically and polished to a thickness of 200 µm at the crystal workshop of the Photon Science Division at DESY. Rotational and translational movements of the two monochromator crystals are induced *via* ex-vacuum motorized stages^1^ that are connected to the in-vacuum copper holder *via* push rods. This construction allows for easy maintenance of the mechanical and electrical parts, but it also requires a careful mechanical adjustment in order to prevent instabilities caused by strain or torque at the air–vacuum interface. Four degrees of freedom are available, *i.e.* pitch (Bragg axis) and roll angle (rotation axis along the incident beam) as well as two translations perpendicular to the beam that determine the position of the incident beam on the crystal.

#### 3.2.2. DuMond diagrams   

**Figure 3:**
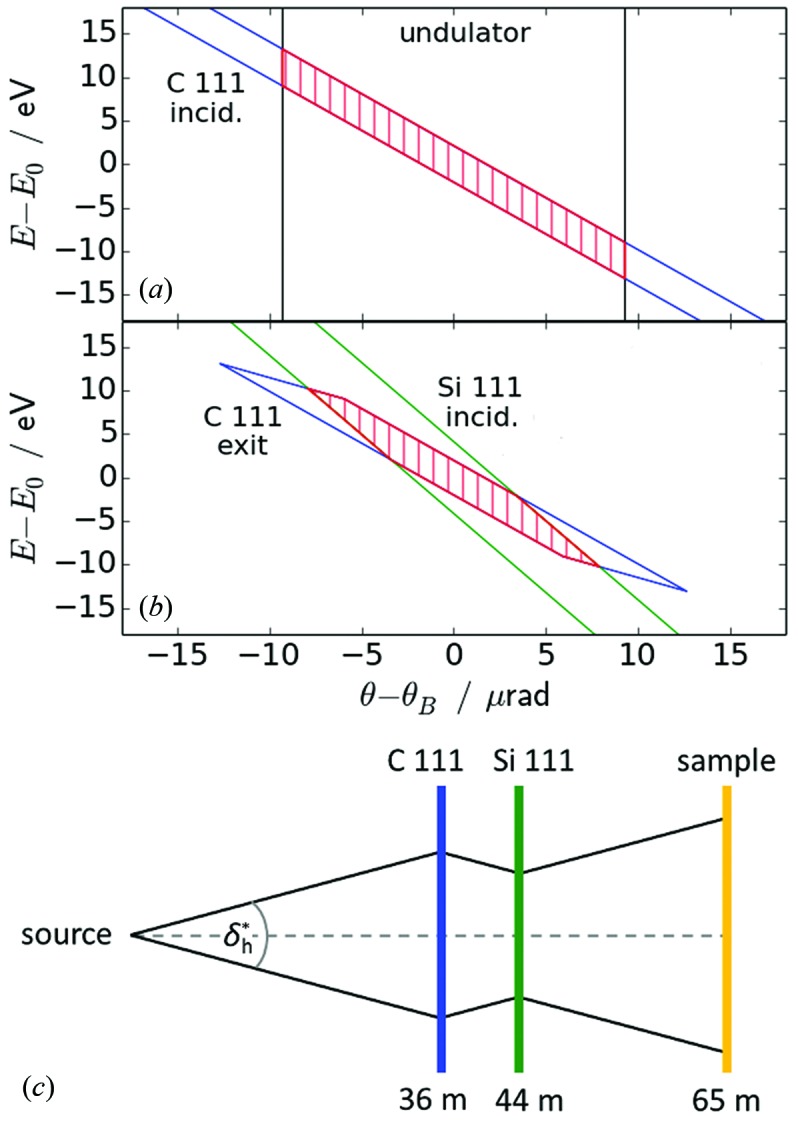
DuMond diagrams of (*a*) the undulator spectrum and the incidence side of the diamond crystal (C 111), and (*b*) the exit side of the diamond crystal and the incidence side of the silicon crystal (Si 111); θ_B_ corresponds to the Bragg angle at the nominal energy *E*_0_ of 60 keV. (*c*) Schematic of the beam geometry to estimate the effective horizontal divergence 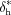 using the intercept theorem (note that 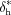 is not identical to the source divergence).

As the two monochromator crystals reflect in the horizontal plane, the vertical beam parameters at any distance are given exclusively by the X-ray source (see §3.3). The horizontal beam parameters on the other hand are determined by the optics. A DuMond diagram (DuMond, 1937 ▶) is a geometrical tool to estimate beam properties such as intensity, divergence and energy spread of the transmitted beam through a multi-crystal monochromator. It is derived by superimposing the average acceptances of the individual crystals in terms of energy (or wavelength) band *versus* diffraction angle for particular reflections. Brauer *et al.* (1995 ▶) derived DuMond diagrams for the case of asymmetrically cut crystals in Bragg and Laue orientation. On this basis, the DuMond diagrams were constructed for the incident and exit sides of the monochromator crystals of P02.1 in Laue geometry as displayed in Fig. 3 ▶ (all values given in FWHM). The starting point on the incidence side is the undulator radiation. In the DuMond diagram, the undulator spectrum is drawn as a rectangle whose width along the θ–θ_B_ axis corresponds to the horizontal divergence and the height along the *E*–*E*_0_ axis to the energy bandwidth. This rectangle represents the range of available photons which may be reflected by the subsequent monochromator crystals. The undulator spectrum was simulated by use of the synchrotron radiation calculation code *SPECTRA* (Tanaka & Kitamura, 2001 ▶). At the position of the power slit at 29 m with an aperture of 1 mm^2^ (see following section), the simulation yielded a horizontal divergence of 19 µrad^2^ and a flux of 9 × 10^12^ photons s^−1^ (0.1% energy bandwidth)^−1^. For the crystals, the Darwin widths were calculated using resources of the *X-ray Server* (Stepanov, 1997 ▶). In the DuMond diagrams, the values of the Darwin widths are multiplied/divided by the square root of the asymmetry factor *b* for the incidence/exit side, respectively, in order to visualize the reflectivity bands (Brauer *et al.*, 1995 ▶). As the diamond 111 Bragg angle for X-rays of 60 keV (2.88°) is very small in comparison with the asymmetry angle (54.74°), *b* is very close to 1 and, hence, the incident and exit widths are very similar.

In comparison with diamond, the reflectivity band of the silicon crystal exhibits a higher slope and a larger Darwin width due to higher electron density and larger lattice spacing of the material. When plotted into the DuMond diagram of the diamond exit side (see Fig. 3*b* ▶), it is obvious that a symmetric silicon 111 crystal does not accept the full ranges in divergence and energy spread that are reflected by the diamond 111 crystal. Instead, the intersection of the two bands yields a hexagon which is marked by the hatched area in Fig. 3(*b*) ▶. For the symmetric Si crystal, the accepted and diffracted beams are identical considering their divergence and energy bandwidth. Therefore, these beam parameters can be estimated from the Si incidence side.

In principle, the divergence and energy spread of the beam produced by a double-crystal monochromator correspond to the intervals of Δθ and Δ*E* spanned by the corners of the intersection area. However, the dispersive arrangement of two differing crystals presented here results in an asymmetric tapered hexagon in the DuMond diagram. The relative area in the tapered corners is relatively small in comparison with the total area of the hexagon, which means that only a small portion of the beam exhibits these extrema of divergence and energy spread. Hence, the overall values of divergence and energy bandwidth are smaller, and they are estimated to be approximately 10 µrad and, respectively, approximately 15 eV, corresponding to Δ*E*/*E* = 2.5 × 10^−4^.

The flux of the monochromatic X-ray beam was estimated as the ratio of the areas of the box-shaped undulator spectrum in Fig. 3(*a*) and the hexagon in Fig. 3(*b*) ▶. This ratio amounts to ∼5%. Taking into account the double Laue configuration, this value is reduced to about 1% because, in approximation, each perfect crystal in Laue geometry only reflects 50% of the incident beam. Relative to the initial flux of the undulator of 9 × 10^12^ photons s^−1^ [this value was calculated using *SPECTRA* (Tanaka & Kitamura, 2001 ▶)], the theoretical flux behind the P02.1 monochromator amounts to 9 × 10^10^ photons s^−1^.

### 3.3. Optical train and beam characteristics   

**Figure 4:**
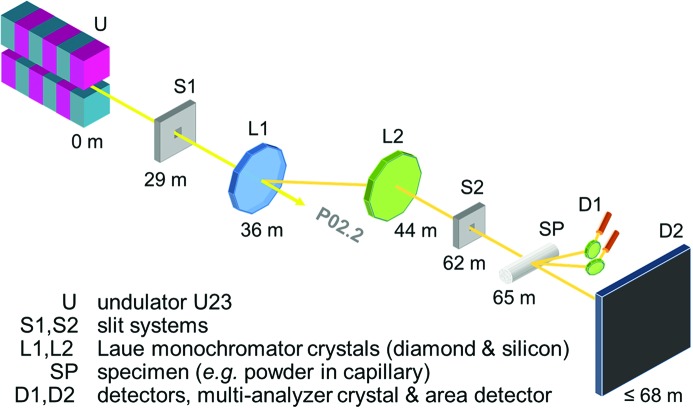
Optical train with the major optical elements of beamline P02.1 and the distances to the undulator source.

Fig. 4 ▶ illustrates the beamline optics for P02.1 from the front-end all the way down to the experimental setup. As mentioned in the previous sections, the undulator is the common source for both P02 experiments. The power slit positioned 29 m downstream from the undulator is set to an aperture of 1 mm × 1 mm. By cutting the spatially outlying portions of the beam, the heat impact on the optical elements is reduced to ∼78 W (calculated using *SPECTRA*). An additional absorber made from 300 µm thin CVD diamond covered with a 50 µm layer of copper is implemented to absorb the low-energy part of the beam and to further reduce the heat load on the P02.1 monochromator by almost 50 W to ∼27 W (calculated using *SPECTRA*). At around 36 m, the diamond crystal is inserted into the beam path as the first monochromator crystal for P02.1 to reflect the 60 keV photons out of the undulator spectrum. The main part of the white beam is transmitted through the thin diamond crystal to the inline station P02.2.

About 8 m further downstream, at a total distance of approximately 44 m from the undulator, the silicon monochromator crystal is positioned. It further conditions the beam as described in §3.2.2 and redirects the beam so that it propagates with only a small angular offset to the sector axis. 100 µm thin diamond fluorescence screens (Degenhardt *et al.*, 2013 ▶) can be inserted behind the two monochromator crystals to track the beam position, and a beam intensity monitor is installed in the experimental hutch to control the beam intensity (see following section). Further optical elements are slit systems and apertures that define the beam profile and clean the beam. This most basic set of beamline optics preserves the naturally high collimation and narrow energy bandwidth of the beam as generated by the X-ray source and the double-crystal monochromator, which are the foundation of diffraction at high angular resolution. As PETRA III is operated in top-up mode at 100 mA with a variation in beam current of 1%, the heat load on the monochromator does not change significantly over time, thus providing one precondition to achieve a very stable X-ray beam.

The beam characteristics that are observed at the sample position at ∼65 m downstream of the undulator are summarized in Table 1 ▶. They were determined at optimized conditions of the insertion device and the optics. During continuous operation, these values may change due to variation of the state of the machine (*e.g.* vacuum conditions), the undulator (*e.g.* demagnetization by the electron beam^3^), and the monochromator (*e.g.* accumulated impurities over operation period). The beam size was measured by fluorescence scans from a 25 µm thin silver wire with an energy-dispersive detector and, more routinely, by slit scans with small aperture. Based on geometrical considerations, the beam size also gives the divergence of the beam. In approximation, the vertical divergence corresponds to the ratio of beam height to distance from the undulator since the beam passes the monochromator practically unmodified. This ratio amounts to ∼9 µrad. The vertical source size of 5 µm (see supporting information) is negligible in comparison with the more than 100 times larger beam size. For the horizontal direction, the divergence is estimated on the basis of the intercept theorem (see Fig. 3*c* ▶). In this construction, a constant effective horizontal divergence 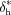 from source to sample position is assumed. The rest of the beam which is accepted by the diamond crystal is lost at the silicon crystal and, hence, does not need to be considered. Taking into account the horizontal source size of 330 µm, the asymmetry factor of around 1.1 and the horizontal beam size of 650 µm measured at the sample position, this approximation yields a horizontal divergence of ∼8 µrad, in good agreement with the conclusions from the DuMond diagrams (see §3.2.2). The photon flux was measured using a calibrated silicon PIN diode^4^ to about 4 × 10^10^ photons s^−1^, and the value was verified by count rate analysis using a CdTe detector with LAMBDA electronics (Pennicard *et al.*, 2011 ▶). This value is lower by a factor of about two than the theoretical estimate from the DuMond diagram, which, however, is based on a theoretical flux calculation for the ideal undulator and does not take into account any crystal imperfections such as mosaicity.

Stability of the monochromator Bragg and roll axes is established by operating the stepper motors in closed loop of encoder readings. This function maximizes the repeatability of the motors during alignment within the required precision range of the order of 10^−5^ degrees. In addition, a piezo actuator is installed on the Bragg axis of the second crystal. It is used for even finer adjustment of the position than possible by the stepper motors and facilitates an active feedback loop with respect to a beam intensity monitor in case that thermal or mechanical drift occurs, *e.g.* during warm-up of the crystal after injection of the beam. Owing to these features, the beam intensity at the sample is stable within about ±2% for more than 60 h.

## 4. Experimental setup   

**Figure 5:**
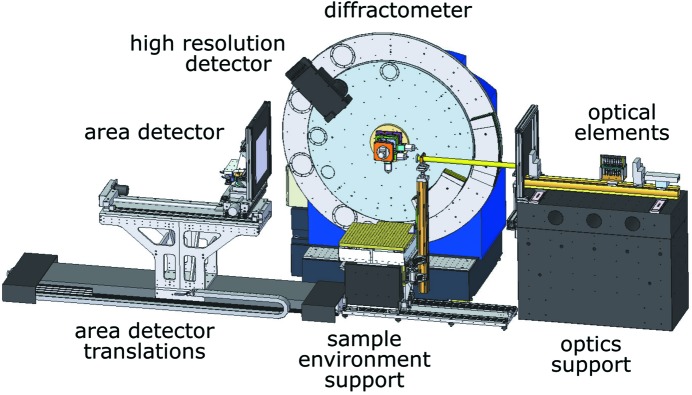
Schematic standard setup of the experimental hutch. In this representation the beam comes in from the right-hand side.

The current standard beamline setup is schematically depicted in Fig. 5 ▶. In this representation the photon beam enters the hutch from the right where a granite support for optical elements is installed. The first optical component is a monitor for beam intensity which is, depending on whether vacuum conditions are required, either an ionization chamber^5^ or a scintillation detector^6^ mounted at a 90° observation angle towards the direct beam. Two slit systems^7^ are implemented, the first of which defines the beam size while the second one acts as the clean-up slit. In addition to the modification of the beam size, the beam intensity can be varied inside the experimental hutch by use of a pneumatically driven attenuator bank consisting of tantalum and tungsten platelets. Further components positioned on the granite support are a fast shutter^8^ that is synchronized with the area detector, and an alignment laser for optical pre-positioning of samples and sample environments. All of the above elements are mounted on a structural rail system for easy movement along the beam axis. At the downstream end of the optics table, a lead screen blocks scattered radiation originating from the optical elements. Further scattering reduction is achieved through a flight tube that connects to the lead screen and ends in a pinhole^9^ setup close to the sample. The position of the pinhole and the length of the flight tube are adjustable for the needs of the specific experimental setup.

The center of the experimental hutch is occupied by the high-resolution diffractometer.^10^ This instrument consists of a goniometer with three concentric circles. Considering the Darwin widths of reflections at 60 keV that are typically in the range of 10^−4^ degrees, a minimum mechanical step size of the diffractometer axes in the range of 10^−5^ degrees is required. In this way, it is possible to record a sufficient number of data points across a peak in order to be able to adequately describe its shape and to perform high-resolution measurements. Detailed specifications with respect to the diffractometer’s accuracy, repeatability, sphere of confusion *etc*. relevant for the high-resolution mode will be described elsewhere (Vad *et al.*, 2015 ▶). The inner circle, the ω axis, carries the sample in case it needs to be positioned in the rotation center of the diffractometer, *e.g.* when a radially scanning detector is used. The load capacity of the ω circle amounts to 50 kg. It usually carries an *xyz* table that provides a travel range of ±25 mm in height and along the beam as well as ±20 mm in the horizontal direction perpendicular to the beam. In this configuration the load capacity of the ω axis is reduced to ∼25 kg, and the lateral distance between the surface of the *xyz* table and the X-ray beam is approximately 100 mm. The two outer circles of the diffractometer (2θ axes) are designed to carry different types of detectors with a maximum weight of 80 kg plus equivalent counter weights. The high-resolution ten-channel multi-analyzer crystal detector [under development, details to be given by Vad *et al.* (2015 ▶)] is installed on the inner 2θ plate while the outer 2θ circle is used for varying setups such as a point detector. In the mid- to long-term, the outermost circle will carry a linear microstrip detector for very fast parallel data acquisition.

In front of the diffractometer, a sample support table is installed to mount samples and sample environments, especially for measurements in transmission geometry by use of the area detector. In particular, *in situ* sample setups that are bulky and/or heavy can be flexibly installed. The table is equipped with a 600 mm × 600 mm mounting plate covered with a 25 mm hole pattern, and exhibits 500 kg load capacity and motorized height adjustment. In addition, the table is manually adjustable on rails along the beam axis to accommodate very large experimental setups. Various standard sample environments that are routinely applied on demand complement the beamline equipment. These include units for thermal treatment such as a hot gas blower for capillaries^11^, cryo streamer systems cooled with liquid nitrogen as well as a continuous-flow cold-finger cryostat flushed with liquid helium or nitrogen^12^. Further devices, *e.g.* for the application of non-ambient temperatures or mechanical force, are available at the Sample Environment and Extreme Conditions Science Infrastructure group^13^. There are ongoing developments to increase the versatility of the sample environments, for instance with respect to temperature range and heating rate as well as sample geometry.

## 5. Fast area detector   

On the downstream side of the sample support table, a motorized support system on rails is installed for the area detector. This detector is routinely used for fast acquisition of diffraction patterns at frame rates up to 15 Hz. It is a model XRD 1621 from PerkinElmer that is characterized by the features given in Table 2 ▶. More details about the detector and its performance can be found in Skinner *et al.* (2012 ▶). As can be seen in Fig. 5 ▶, the area detector is mounted on two separate translations parallel to the beam axis, one on the floor and the other one on the frame that sits on the bottom translation. The translation table system was designed to offer great flexibility in moving the detector either very close to the sample, regardless of the position of the sample environment table, or as far back as possible close to the downstream wall of the hutch. The resulting range of obtainable sample-to-detector distance (SDD) amounts to approximately 200–3000 mm. An additional horizontal translation perpendicular to the beam axis enables a motorized movement to off-center the detector by ∼400 mm in order to increase the accessible *Q* range for any given SDD. It must, however, be considered that such horizontal detector displacement reduces the usable azimuthal range for integration of the two-dimensional diffraction pattern and, thus, the average counting statistics.

**Figure 6:**
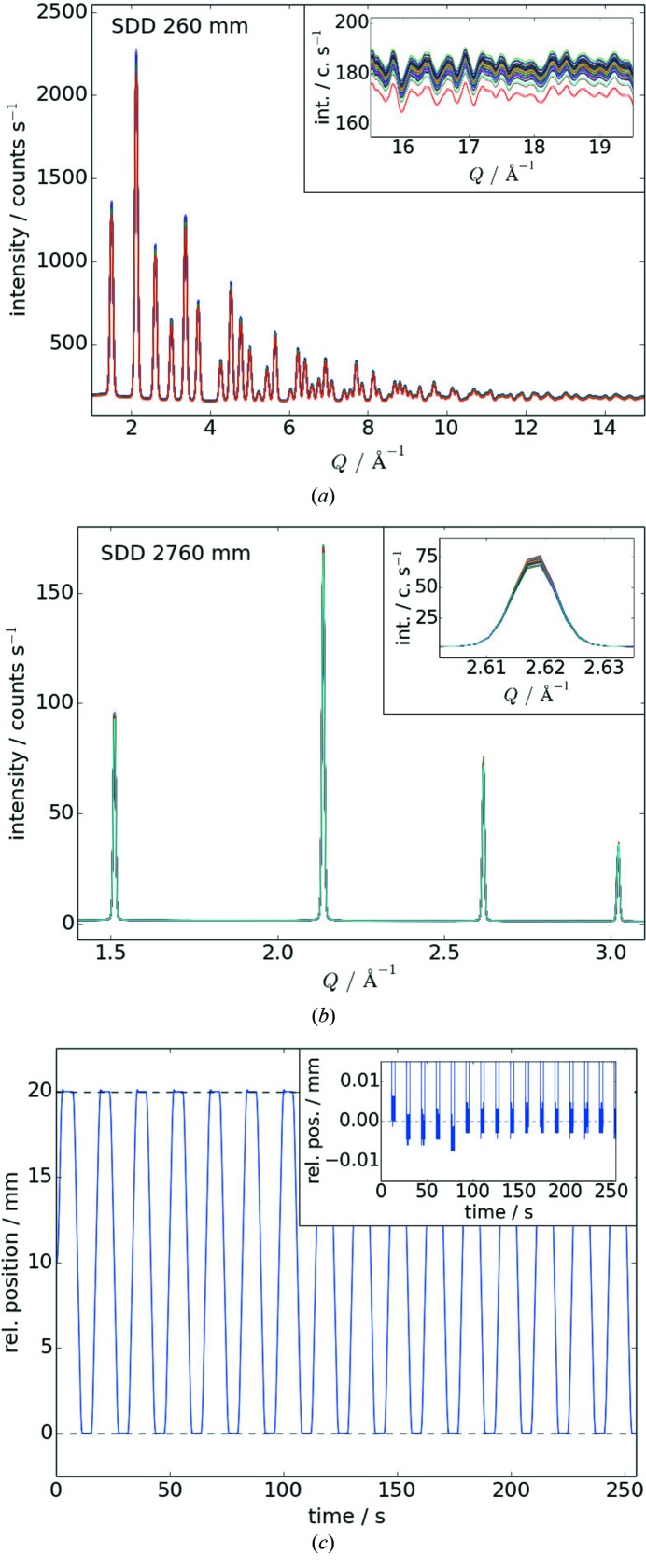
Repeatability of the area detector shown by means of (*a*, *b*) XRD patterns of LaB_6_ collected at short and long sample-to-detector distance (SDD), respectively, and (*c*) laser interferometer measurements of repeated back-and-forth movement with a total travel range of 20 mm within the detection range of the interferometer. Each inset scales up the data in intensity and relative position, respectively, to emphasize the corresponding small errors in diffraction angle and position.

The described setup meets the technical requirements to collect data up to a high *Q* range at short SDD or at relatively high angular resolution at long SDD. At all available SDDs, it is possible to observe anisotropic effects such as texture, preferred orientation and spatially varying lattice parameters in stressed or strained samples. All these techniques can be combined by moving the area detector to different SDDs during the measurement of a single specimen. In this context, the reliability of the detector positioning system is of utmost importance for data quality. Hence, the repeatability of the detector translations along the beam was tested in a series of exposures of a LaB_6_ standard (NIST 660a) filled into a 0.8 mm-diameter capillary. During this measurement, the detector was moved forward and backward (with and without backlash) over the entire travel range in steps of 125 mm. Representative integrated diffraction patterns^14^ from these measurements are shown in Figs. 6(*a*) and 6(*b*) ▶. Regardless of little intensity fluctuation, the recorded reflections coincide perfectly in *Q* and thus demonstrate that the repeatability is better than the angular resolution of the area detector. In order to quantify the absolute positioning error, laser interferometer^15^ measurements were carried out. The corresponding results displayed in Fig. 6(*c*) ▶ show that the detector arrives at the desired position each time within a precision of ∼10 µm. Consequently, the area detector setup allows for calibrating the detector at specific SDDs before the experiment and reproducibly moving it back to the calibrated positions, provided that no mechanical interference with the setup occurs during stable beam operation.

**Figure 7:**
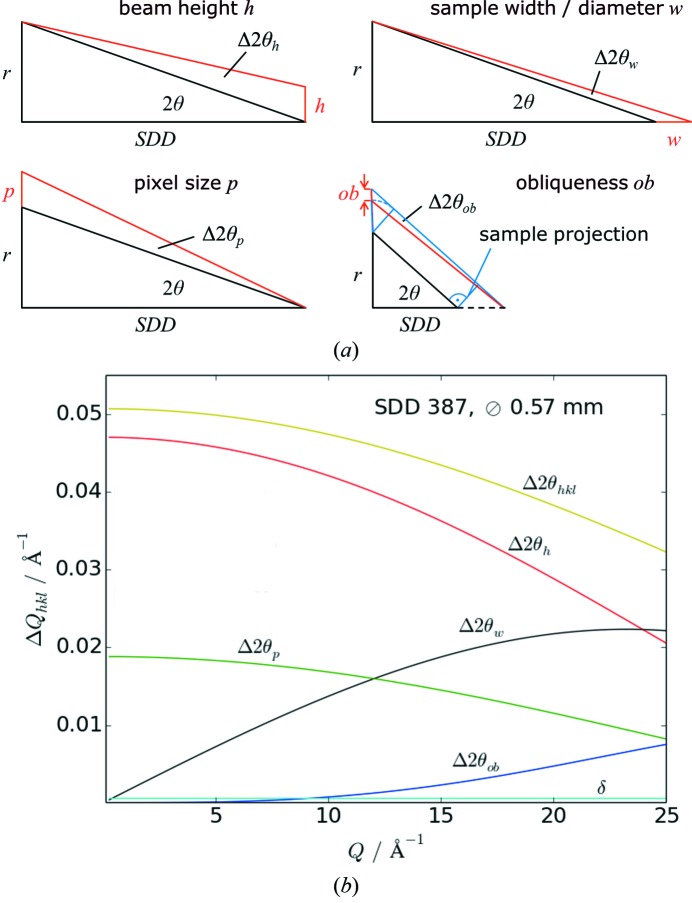
(*a*) Simplified geometrical construction of the different contributions to peak broadening of reflections on the area detector. In the calculation, all parameters are expressed in terms of the diffraction angle 2θ, the respective sample-to-detector distance SDD, the given sample dimensions and beam height. (*b*) Individual contributions to peak broadening and the Gaussian convolution according to equation (1) for a given measurement geometry (sample-to-detector distance 387 mm, capillary diameter 0.57 mm).

**Figure 8:**
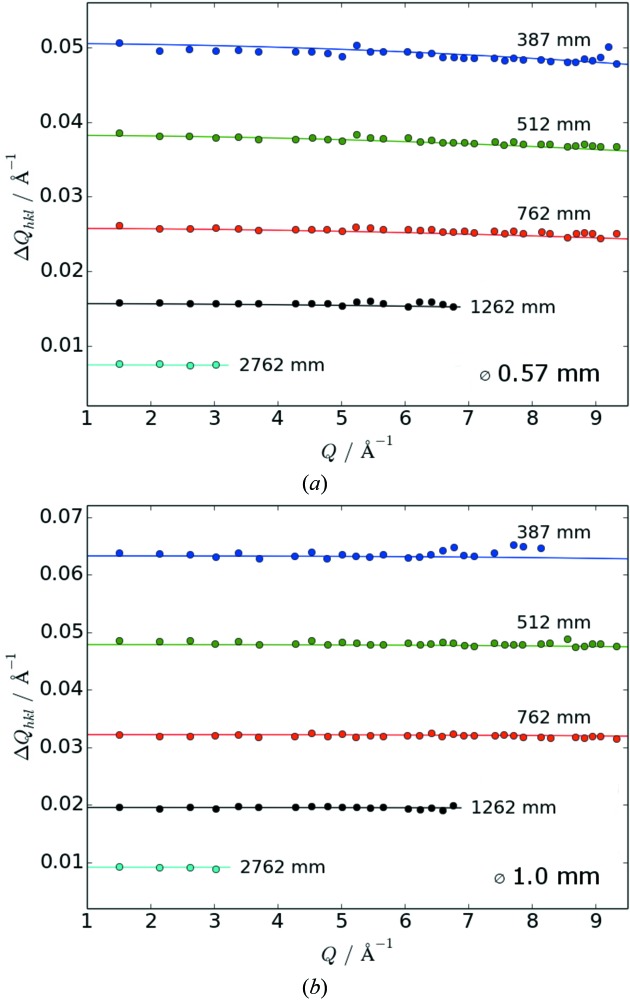
Resolution function of the area detector for two exemplary capillaries of diameters 0.57 and 1 mm, respectively, filled with LaB_6_ (NIST standard 660a); dots represent the experimental data, lines the calculated values according to equation (1). At the short sample-to-detector distance (SDD), the diffraction patterns obtained for the thicker capillary (Fig. 6*a* ▶) show considerable overlap at higher *Q* values (*Q* > 8 Å^−1^) so that determination of the FWHM was not possible.

Apart from the chosen SDD, several other experimental parameters have to be taken into account in order to achieve optimum data quality for a given measurement task. In the following, the major factors influencing the peak width of the reflections and therefore limiting the resolution are discussed. Obviously the area detector specifications, *i.e.* pixel size *p* and point spread function, affect the resolution. Additional broadening is caused by the obliqueness *ob* of the detector, *i.e.* its fixed alignment perpendicular to the direct beam instead of being rotated by 2θ to provide normal incidence to the diffracted beam^16^ The most crucial role, however, play the sample and beam dimensions, *e.g.* capillary diameter or thickness of a flat plate sample *w* in transmission geometry, and beam height *h*. Based on purely geometrical considerations as depicted in Fig. 7(*a*) ▶, the instrumental resolution function of the area detector setup was calculated as a function of the diffraction angle 2θ for varying SDD as a Gaussian convolution of the individual contributions according to 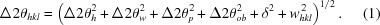
Here, Δ2θ_*hkl*_ is the total full width at half-maximum (FWHM) of a reflection *hkl* resulting from broadening by the beam height Δ2θ_*h*_ 17, the capillary diameter or sample thickness Δ2θ_*w*_, the pixel size Δ2θ_*p*_ (including point spread function), the obliqueness Δ2θ_*ob*_, and the divergence δ (see Table 1 ▶), which all add on to the intrinsic width of the reflection *w*_*hkl*_. At 60 keV, the intrinsic widths of the sample reflections of an unstrained microcrystalline powder are negligible in comparison with the detector- and beam-related parameters. Fig. 7(*b*) ▶ depicts the individual contributions from equation (1) (neglecting *w*_*hkl*_) and their convolution over a wide range of *Q* = 4πsinθ/λ for a representative sample geometry. In Fig. 8 ▶, the theoretical curves for chosen SDDs converted to *Q* range are plotted together with the FWHM values extracted from diffraction patterns of standard measurements (LaB_6_ NIST 660a, 0.57 and 1.0 mm capillary diameters and full beam height). It is obvious that the resolution changes with the SDD by roughly one order of magnitude over the available range of distances. It is also evident that the choice of capillary diameter, which *de facto* determines the maximum effective beam height, has a major impact on the resolution which is most prominent at shorter SDDs. When spinning of the capillary is not perfect, the wobble increases the effective capillary size. On the basis of the presented data, the preparation of an experiment should always include, whenever possible, a deliberate choice of the sample and beam dimensions which are appropriate for the desired resolution in reciprocal space. If higher angular resolution is required, the multi-analyzer crystal detector will offer a resolution higher by at least two orders of magnitude but considerably longer collection times (minutes to hours).

## 6. Further developments   

During the first two and a half years of user operation, beamline P02.1 has been ramping up its capabilities to serve various facets of powder diffraction that exploit the high angular collimation as well as the high energy and the high flux of the X-ray beam. User communities who study, for example, nano- and engineering materials frequently have the demand for small beam sizes from several tens to a few hundreds of micrometers. This setting can currently only be achieved by slitting down the beam at a significant loss of intensity. In order to enhance the beamline performance for those PXRD experiments that require small beam sizes, the installation of focusing optics in the form of compound refractive lenses is being prepared. The lens changer setup will also feature a slit system to vary the beam size in the optics hutch, *i.e.* well upstream of the current beam-defining aperture in the experimental hutch, which will significantly reduce the stray radiation at the sample position.

In addition, automated sample-changer systems will be installed to facilitate high-throughput data collection. This option will be particularly effective when acquiring images on the fast area detector. In this case, the manual exchange of a sample (including the procedure to set the hutch interlock) may take as much time as a single exposure for a sample. A multi-axes industrial robot will be integrated to handle series of capillaries or other samples that are to be measured at ambient conditions or at high or low temperatures applied by the hot-gas blower or cryo-streamer (§4). For other sample types, customized sample changers that use one or more motorized translations or rotations are already in use, but more will be developed as needed.

Upgrades on the detector side include a motorized vertical translation of at least 200 mm as a feature for the available area detector. It will enlarge the accessible reciprocal space for upward scattering experiments which is very important for horizontally positioned samples in reflection geometry such as thin films. Eventually, the integration of new detectors using high-*Z* sensor materials will improve the time-resolution of the beamline beyond 15 Hz as provided by the current area detector. Linear microstrip detectors for massive parallel data collection are currently only available based on silicon as the photon-counting material (Bergamaschi *et al.*, 2009 ▶). These systems are not suitable for use at P02.1 because of their low quantum efficiency at 60 keV. While a point detector equipped with a CdTe diode^18^ is available at the beamline, pixel detectors on the basis of Ge, GaAs or CdTe semiconductors are still in the prototype phase (Pennicard & Graafsma, 2011 ▶; Pennicard *et al.*, 2011 ▶). Once this technology is ready to be implemented in a microstrip detector array that covers a large angular range (*e.g.* 2θ = 60°), the installation of such a device will be pursued. 

## 7. Experimental highlights   

Below we point out research activities that have been carried out at beamline P02.1 during the early stage of user operation. These examples cover the three main techniques available at P02.1, *i.e.* high-angular-resolution measurements, total scattering, and *in situ* studies, which were applied to different scientific disciplines.

**Figure 9:**
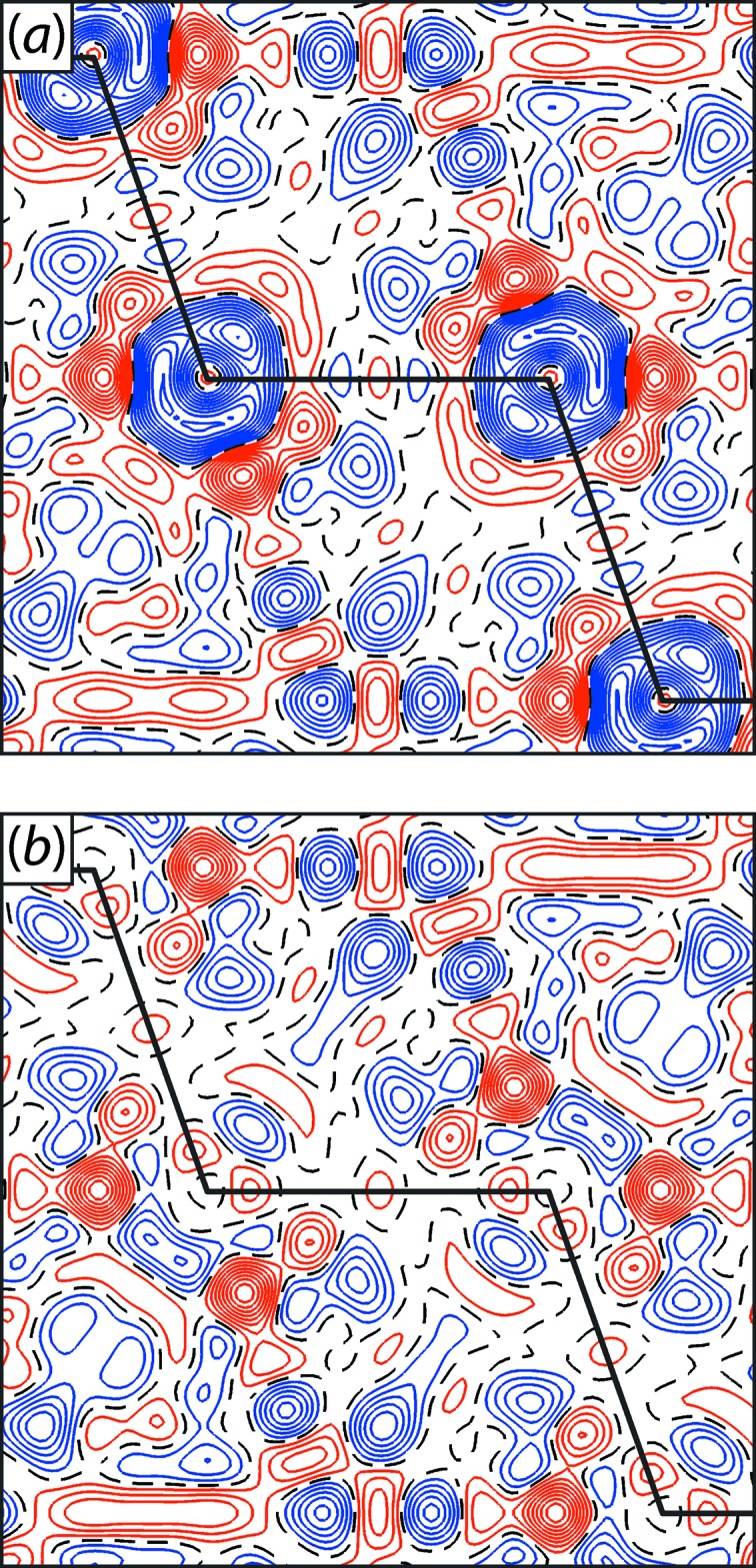
Contraction of the innermost electron density in diamond revealed by residual plots based on multipolar modeling using (*a*) a frozen and (*b*) a flexible core description. Positive (red), negative (blue) and zero (dotted black) contour lines are drawn with a step width of 0.01 e Å^−3^. The solid black line represents the C—C bonding chain (Bindzus *et al.*, 2014 ▶).

The high collimation of the high-energy X-ray beam was exploited for the determination of charge density distributions (CDDs) from powder diffraction data (Bindzus *et al.*, 2014 ▶; Straasø *et al.*, 2014 ▶). The CDD of molecules and compounds holds valuable information on their electronic structure and chemical bonding. Usually, CDDs are calculated from structure factors derived by single-crystal measurements. However, powder diffraction offers various advantages, *e.g.* easier sample preparation, shorter data acquisition times and avoiding errors due to extinction and scaling of the necessary series of exposures (Svendsen *et al.*, 2010 ▶). Moreover, it is essential to advance this technique as it can be applied in many cases when single-crystal measurements are not a viable option. An all-in-vacuum diffractometer (Straasø *et al.*, 2013 ▶) was used for the experiments at P02.1 which suppresses background scattering to the minimum of Compton scattering from the sample and sample holder. This setup is equipped with an image plate in Debye–Scherrer geometry. Since the whole instrument is movable, it is an effective alternative to a multi-analyzer crystal detector installation, offering data with comparable statistics and signal-to-noise ratios at notably shorter acquisition times. At P02.1, benchmark data were collected up to sinθ/λ ≥ 2 where the ratio of incoherent Compton scattering to the total signal is close to 1, in particular for light elements such as carbon. From the extracted structure factors, CDD maps were calculated with unprecedented accuracy, *e.g.* for the reference material diamond (Fig. 9 ▶). The results revealed even the minuscule contribution of core electron deformation due to chemical bonding which had previously only been predicted theoretically (Fischer *et al.*, 2011 ▶). This technique is undergoing further technical development and is being expanded to study the CDDs of higher-*Z* elements and more complex compounds.

Crystal structure solution and refinement from powder diffraction data by the Rietveld method is a common task for crystallographers and chemists. In fact, the quantitative analysis of complex structures usually involves a great number of parameters to be refined when the classic atomic coordinates model is applied. In order to increase the significance of the refinements, *e.g.* to describe octahedral rotations, other naturally constrained models such as rigid-body approaches can be very effective (Dinnebier, 1999 ▶). In a case study on the cubic to monoclinic phase transition of the double salt Mg(H_2_O)_6_RbBr_3_ (Dinnebier *et al.*, 2008 ▶), four different refinement models were compared using readily available Rietveld analysis software. Corresponding *in situ* data were obtained at P02.1 during thermal treatment in the temperature range 30–149°C, covering the phase transition at 138°C. The area detector was employed for data acquisition. Parametric as well as sequential fits were performed on the basis of models refining atomic coordinates, traditional rigid-body parameters, purely displacive symmetry modes (see, for example, Campbell *et al.*, 2007 ▶) and rigid-body rotational symmetry modes (Müller *et al.*, 2010 ▶). While comparable absolute values were achieved in all four models at constant temperature, the reduced number of variables in the three constrained models yielded lower estimated standard deviations in comparison with the unconstrained model of atomic coordinates. The equivalent results verified the applicability of the new model of rigid-body rotational symmetry modes to the investigated material class, and it will reveal its full potential of structural simplification in the analysis of more complex structures.

As described in detail in §5, there are many parameters that determine the observed peak width of the reflections when acquiring data on the area detector. In a given *in situ* setup that is dedicated to a particular kind of experiment, there are, however, limitations to the choice of sample and beam parameters, but they do not necessarily affect the outcome of the experiment. This was demonstrated for the case of battery materials that are studied *operando* in customized electrochemical cells (Herklotz *et al.*, 2013 ▶). Lithium ion batteries are one of the key components in ever more powerful mobile electronics. This aspect drives global research and development to steadily improve their performance with respect to power density, efficiency, durability, charging rate and safety *etc.* (see, for example, Whittingham, 2011 ▶). A multitude of lithium compounds such as ternary oxides are used for different constituents of the batteries (Goodenough & Park, 2013 ▶) along with a large set of other functional materials such as metals (counter electrode, charge collector) and polymers or glass (separator, sealing, *etc.*). *In situ* powder diffraction allows tracking the structural changes during electrochemical cycling and, thus, helps to identify related mechanisms of battery degradation. The signal-to-noise ratio of the weakly scattering lithium compound is unfavorable in the highly complex electrochemical cell. In addition, high angular resolution is required to separate the phases and to perform microstructure analysis, for instance to derive crystallite sizes. Furthermore, high-energy X-rays at high flux are best suited in order to penetrate the cell without considerable absorption and to observe rapid phase transitions during charge and discharge processes. The survey carried out at P02.1 reinforces the outstanding performance in all these respects for the *operando* investigation of lithium batteries in a customized test cell design. A time-resolution of 150 ms was achieved which opens up the possibility to operate a multi-cell holder. This holder can carry several operating electrochemical cells that are sequentially measured in a loop. Owing to the short collection time, the number of samples measured during the same run may be substantially increased beyond four cells as in the current setup. At a moderate sample-to-detector distance of 1200 mm, the obtained angular resolution using the area detector was found to be sufficient to extract apparent crystallite sizes from the diffraction data while simultaneously recording a considerable range in *Q* space. New insights into the structure of disordered (intermediate) phases and the amorphous components of electrochemical cells will be gained by applying X-ray total scattering methods.

**Figure 10:**
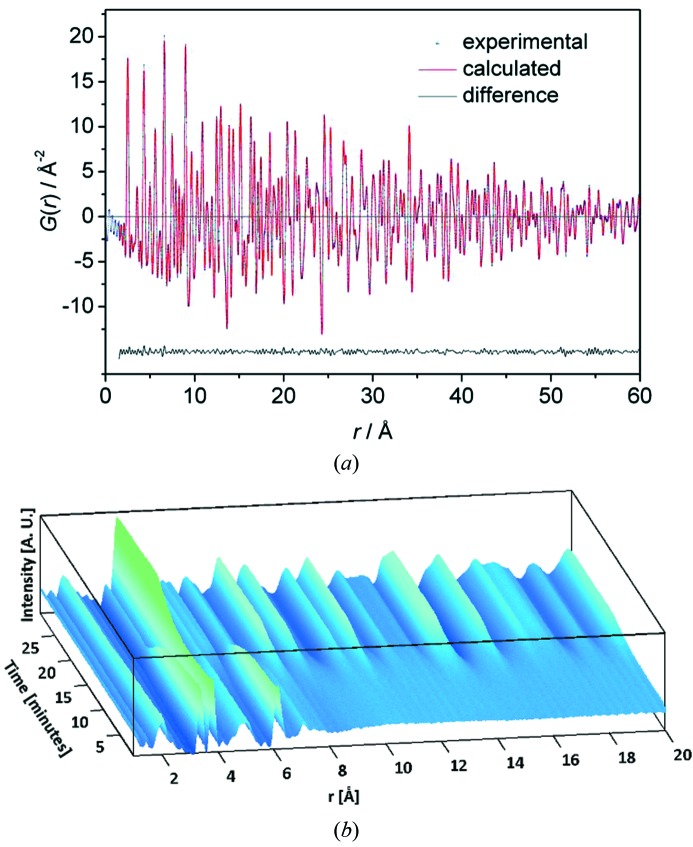
(*a*) Benchmark pair distribution function (PDF) from a nickel standard sample (SDD = 307 mm, *Q*_max_ = 20 Å^−1^, *R*_w_ = 3.8% for the *r* range 1.5–60 Å; data taken within 5 s exposure time on the area detector). (*b*) *In situ* PDFs from the synthesis of tungsten oxide nanoparticles by hydrothermal synthesis with a time-resolution of 1 s (Saha *et al.*, 2014 ▶).

X-ray total scattering analysis is a more and more widely used technique that combines the evaluation of diffraction data in reciprocal and real space to deduce the global as well as the local structure of a sample (see, for example, Egami & Billinge, 2012 ▶; Proffen & Kim, 2009 ▶). A precondition to obtaining high resolution in real space is that diffraction data are measured with good counting statistics up to high momentum transfer *Q*. Fig. 10(*a*) ▶ shows a benchmark pair distribution function (PDF) of nickel powder in a 1 mm diameter capillary collected at P02.1 within 5 s at a sample-to-detector distance of 307 mm, the detector being off-centered by 175 mm. Using the *PDFgui* program (Farrow *et al.*, 2007 ▶) with a *Q*_max_ setting of 20 Å^−1^, the data set was fitted to the model of the 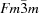 crystal structure by a remarkably low error value *R*_w_ = 3.8% over the distance range 1.5 ≤ *r* ≤ 60 Å. A particular benefit of the PDF representation of diffraction data is the possibility to directly follow changes in the nearest-neighbor distances of chemical species during the course of a reaction or any other kind of transformation. This statement holds regardless of the chemical or crystallographic nature of the involved compounds, as long as high-quality data (low noise at high *Q*) are obtained. The significance and efficiency of X-ray total scattering in the *in situ* studies of chemical reactions was recently reviewed for the case of solvothermal synthesis of energy materials (Jensen *et al.*, 2014 ▶). Saha *et al.* (2014 ▶) reported on the results of Rietveld refinements in real and reciprocal space for the *in situ* hydrothermal synthesis (Becker *et al.*, 2010 ▶) of tungsten oxide nanoparticles recently performed at P02.1. By evaluating the derived PDFs for different stages of the reaction, it was shown that the reaction mechanism involves a kinetically controlled reorientation of the WO_6_ octahedra in the precursor cluster molecule before the formation of crystalline nanoparticles of hexagonal crystal structure set in. The time resolution of the experiment was 1 s over the whole duration of the experiment (30 min); a representation of corresponding PDFs *versus* time is displayed in Fig. 10(*b*) ▶. These new insights into the reaction pathway on a molecular level, *i.e.* the structural rearrangement during the rate-limiting step, led to a more profound understanding of polymorphism of WO_3_ nanoparticles produced by varying preparation methods on different time-scales down to fractions of a second. Based on this kind of knowledge, it will be possible to improve the ability to control the characteristics of wet-chemically synthesized nanoparticles and to more efficiently tailor them for particular industrial applications.

## 8. Summary and outlook   

The High Resolution Powder Diffraction Beamline P02.1 at PETRA III is a versatile instrument for structural studies in materials science, crystallography and chemistry. This paper describes the beamline’s scope and illustrates the current capabilities by showing some highlight experiments from various topics in basic and applied research carried out during the first two and a half years of user operation. Details are given on the design of the optics and the experimental setup. The performance of the installed area detector is characterized with respect to resolution and repeatability of its positioning system. The influence of varying sample and beam geometries on the peak widths of the observed reflections are described by an analytical function which serves as a guide for users to estimate the expected resolution for their samples and to choose optimum beam settings. By upgrading the beamline with focusing optics and a high-*Z* material strip detector that covers a wide range in diffraction angle, the capabilities of P02.1 in terms of spatial and time resolution will advance within the next years.

## 9. Related literature   

The following references are mentioned in the Supporting Information: PETRA III (2004 ▶); Rodgriguez-Carvajal (2001 ▶).

## Supplementary Material

Machine and undulator parameters; Rietveld refinements and instrumental parameters for area detector. DOI: 10.1107/S1600577515002222/co5063sup1.pdf

## Figures and Tables

**Table 1:** Beam characteristics (in terms of FWHM) at the sample position (65m) in the current layout (without focusing); theoretical values are marked by an asterisk

Beam size, H V	650m 600m
Divergence, H V	8rad 9rad
Energy bandwidth	
absolute	16eV*
relative	2.5 10^4^*
Photon flux	4 10^10^photons s^1^

**Table 2:** Characteristics of the area detector available at P02.1 PerkinElmer XRD 1621 amorphous silicon flat-panel detector.

Active area	400mm 400mm
Pixel size	200m 200m
Pixel count	2048 2048
Dynamic range†	16 bit
Maximum frame rate	15Hz
Readout time	67ms
Point spread function	1.1 pixels

† Of the analog-to-digital converter (ADC).

## Footnotes

1 Huber Diffraktionstechnik, Rimsting, Germany, and OWIS GmbH, Staufen, Germany.

2 The divergence is not determined by the aperture, as the entire central cone of the beam passes the slit of 1 mm × 1 mm at 29 m. The naturally low divergence is a direct result of the low emittance of the electron beam.

3 This effect was described by Vagin *et al.* (2014 ▶). The P02 undulator was reconditioned at the end of 2014.

4 Type Hamamatsu S3584-06, on loan from the Advanced Photon Source.

5 Type ADC Ion Chamber 105-0000-2, air-filled, used with Keithley model 428 current amplifier.

6 Cyberstar (FMB Oxford Ltd, Oxford, UK) systems comprising detector heads equipped with YAP or NaI scintillator crystals, used with polyimide scattering foil at 45° in the beam.

7 Model JJ X-ray IB-C30-HV.

8 Electro-Optical Products Corp., Ridgewood, New York, USA.

9 Platinum, thickness 1 mm, diameters ranging from 1 to 1.5 mm, manufacturer Ladd Research Industries, Williston, Vermont, USA.

10 Rotary Precision Instruments UK Ltd, Bath, UK.

11 Type Cyberstar Hot Air Blower.

12 Models from Oxford Cryosystems and Cryo Industries of America.

13 See web page at http://photon-science.desy.de/p3_sample_environments.

14 Integration by use of the program *FIT2D* (Hammersley *et al.*, 1996 ▶).

15 Type Polytec single-point vibrometer QFV505.

16 This effect does not occur when the detector has the shape of a spherical shell with radius SDD.

17 Note that in the case of capillary measurements *h*_max_ ≤ *w* (capillary diameter).

18 Model Amptek XR-100T.
