# Supplementary material for: Beamline P02.1 at PETRA III for high-resolution and high-energy powder diffraction
Source: J Synchrotron Radiat. 2015 Apr 14;22(Pt 3):675–87. doi: 10.1107/S1600577515002222 (PMC4416682; doi:10.1107/S1600577515002222)
Supplement: Supplementary file 1 [file s-22-00675-sup1.pdf]

JOURNAL OF SYNCHROTRON RADIATION

Volume 22 (2015)

Supporting information for article:

Beamline P02.1 at PETRA III for high-resolution and high-energy powder diffraction

Ann-Christin Dippel, Hanns-Peter Liermann, Jan Torben Delitz, Peter Walter, Horst Schulte-Schrepping, Oliver H. Seeck and Hermann Franz

Table of Contents

S1. Machine and undulator parameters.

S2. Rietveld refinements for determination of instrumental resolution of the area detector.

IUCr

Journal of Synchrotron Radiation supporting information

S1. Machine and undulator parameters

Table S1.A: Selected machine parameters (complete list on http://photon-science.desy.de/facilities/petra_iii/machine/parameters/index_eng.html)

circumference	2304 m
energy	6.08 GeV
beam current	100 mA (top-up mode)
bunch number	960 / 40
RF frequency	500 MHz
horizontal emittance	1 nmrad
vertical emittance	10 pmrad
horizontal high-β function (5m undulator)	20 m rad^{-1}
vertical high-β function (5m undulator)	2.4 m rad^{-1}

Table S1.B: Selected undulator parameters (from Barthelmess *et al.* 2008 and calculated using the PETRA III Technical Design Report).

device	U23
type	planar, ex-vacuum
magnet material	Ni-Fe-B
device length	2 m
period length	23 mm
minimum gap	9.5 mm
gap @ 60 keV	~ 10 mm
peak magnetic field	0.61 T
total power	1.7 kW
on-axis power density	71 kW $mrad^{-2}$
high-β source size @ 60 keV, H × V (1σ)	127 × 5.0 μm^2
high-β source divergence @ 60 keV, H × V (1σ)	8.2 × 2.3 μrad^2

S2. Rieetveld refinements for determination of instrumental resolution of the area detector

Figure S2.A: Exemplary Rietveld refinement plots and parameters for a PXRD measurement of LaB_6 filled into a capillary of 0.5 mm at a sample to detector distance of 510 mm. The refinement was carried out using the FullProf Suite (Rodriguez-Carvajal 2001), using Thompson-Cox-Hastings pseudo-Voigt peak shape and Chebychev polynomial background type with 5 coefficients. Left: full pattern; right: zoom into higher 2θ region.

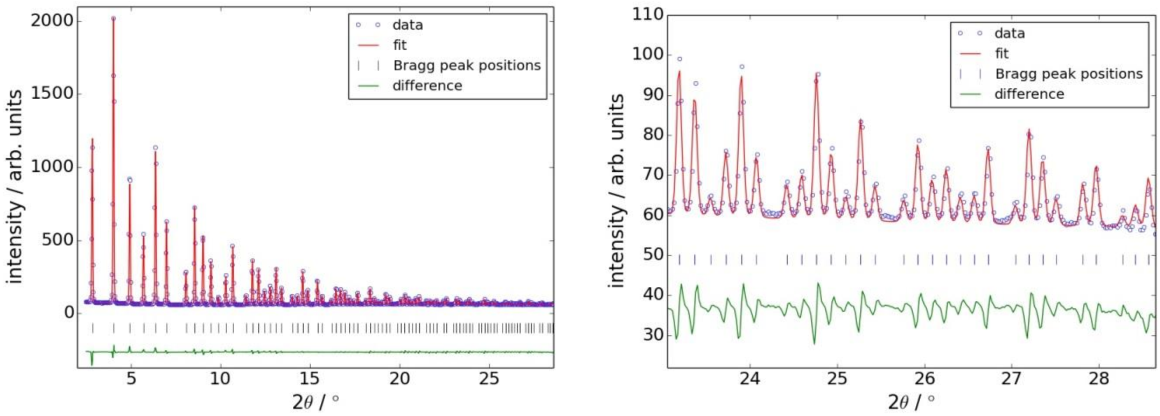

refined parameters:		agreement factors:	
a	4.154626	R_p	6.26
zero shift	-0.00324	R_{wp}	6.08
scale	0.627 E-05	R_B	2.30
B_{iso} (La)	0.318(1)	R_F	2.81
B_{iso} (B)	0.277(8)	χ^2	0.0876
U	0.0356(6)		
V	-0.0076(6)		
W	0.0035(7)		
Y	0.0114(9)		

Table S2: Instrumental parameters from Rietveld refinement of data obtained in measurements of LaB_6 filled into capillaries of 0.57 mm (upper values in regular) and 1.0 mm (lower values in *italics*) diamenter, respectively. Rietveld refinements were carried out using the FullProf Suite (Rodgriguez-Carvajal 2001)), using Thompson-Cox-Hastings pseudo-Voigt peak shape and Chebychev polynomial background type with 5 coefficients..

sample to detector distance (SDD)	U	V	W	Y
385	0.0542(3)<br>*0.0488(4)*	-0.0123(4)<br>*-0.0088(9)*	0.0061(4)<br>*0.0098(4)*	0.0114(9)<br>*0.0057(0)*
510	0.0356(6)<br>*0.0417(5)*	-0.0076(6)<br>*-0.0070(7)*	0.0035(7)<br>*0.0057(0)*	0.0086(3)<br>*0.0048(4)*
760	0.0204(9)<br>*0.0228(4)*	-0.0037(8)<br>*-0.0037(1)*	0.0016(4)<br>*0.0025(8)*	0.0057(4)<br>*0.0038(5)*
1260	0.0223(8)<br>*0.0100(2)*	-0.0026(2)<br>*-0.0011(6)*	0.0006(4)<br>*0.0009(3)*	0.0035(2)<br>*0.0027(2)*
2760	0.0103(4)<br>*0.0049(8)*	-0.0008(3)<br>*-0.0005(3)*	0.0001(4)<br>*0.0002(2)*	0.0017(5)<br>*0.0015(6)*

References

Barthelmess, M., Englisch, U., Pflüger, J., Schöps, A., Skupin, J. & Tischer, M. (2008). *Proceedings of the 11th European Particle Accelerator Conference (EPAC08)*, 2320.

PETRA III Technical Design Report, *DESY 2004-035*, available on http://petra3-project.desy.de/general/tdr/index_eng.html.

Rodgriguez-Carvajal, J. (2001). *Commission on Powder Diffraction (IUCr) Newsletter*, **26**, 12-19.
